# Prospects and perils in the geospatial turn of conservation

**DOI:** 10.1111/cobi.70145

**Published:** 2025-09-09

**Authors:** Jocelyne Shimin Sze, Laura Aileen Sauls

**Affiliations:** ^1^ Institut de Ciencia i Tecnologia Ambientales (ICTA‐UAB) Universitat Autonoma de Barcelona Bellaterra Spain; ^2^ Global Affairs Program George Mason University Fairfax Virginia USA

**Keywords:** big geospatial data, data justice, decision‐making, GIS, global maps, Justicia de datos, macrodatos geoespaciales, mapas globales, SIG, toma de decisiones, 地理空间大数据, 全球地图, GIS, 决策, 数据正义

## Abstract

Conservation has embraced advances in big data and related digital technologies as key to preventing biodiversity loss, especially in the identification of areas of conservation priority based on spatial data, which we call the big geospatial data turn. This turn has led to the proliferation of useful methods and tools, including global geospatial maps. But these methods may also undermine moves toward rights‐based and inclusive conservation approaches that consider plural values and perspectives. We built on the burgeoning literature to call for greater attention to be paid to the datasets, methodological choices, and the assumptions global mapping for biodiversity conservation is based on. In increasingly prioritizing the use of big geospatial data, conservation professionals risk forgetting that maps show only partial information and limit the diversity of ways of seeing and representing the world. Big geospatial data collected through remotely sensed technologies must still be situated in time and place and provided with appropriate political–economic and sociocultural contexts. Further, global mapping efforts remain primarily the purview of Global North researchers, even given the push to make data open access. Instead of uncritically calling for more data, we urge conservationists to contextualize and situate big geospatial data carefully so as to build a field that achieves socially just and ecologically effective conservation outcomes.

## Abstract (es)

Prospectos y peligros en el giro geoespacial de la conservación

**Resumen:**

La conservación ha adoptado los avances en el ámbito de los macrodatos y las tecnologías digitales relacionadas como elementos clave para prevenir la pérdida de biodiversidad, especialmente en la identificación de áreas prioritarias para la conservación basadas en datos espaciales, lo que denominamos el gran giro geoespacial. Este giro ha dado lugar a la proliferación de métodos y herramientas útiles, incluidos los mapas geoespaciales globales. Sin embargo, estos métodos también pueden detener los avances hacia enfoques de conservación inclusivos y basados en los derechos que tienen en cuenta valores y perspectivas plurales. Nos basamos en la creciente bibliografía para solicitar que se preste más atención a los conjuntos de datos, las elecciones metodológicas y los supuestos en los que se basa la cartografía global para la conservación de la biodiversidad. Al dar cada vez más prioridad al uso de los macrodatos geoespaciales, los profesionales de la conservación corren el riesgo de olvidar que los mapas solo muestran información parcial y limitan la diversidad de formas de ver y representar el mundo. Los macrodatos geoespaciales recopilados mediante tecnologías de teledetección deben seguir situándose en el tiempo y el espacio y proporcionarse en contextos políticos, económicos y socioculturales adecuados. Además, los esfuerzos de cartografía global siguen siendo principalmente competencia de los investigadores del Norte Global, incluso a pesar del impulso para que los datos sean de libre acceso. En lugar de pedir más datos sin sentido crítico, instamos a los conservacionistas a contextualizar y situar cuidadosamente los macrodatos geoespaciales para construir un campo que logre resultados de conservación socialmente justos y ecológicamente eficaces.

## 保护领域地理空间转向的前景与风险

【摘要】 在保护生物学领域, 人们已将大数据及相关数字技术的进步视为遏制生物多样性丧失的关键, 特别是在基于空间数据确定优先保护区方面, 我们将这一转变称为“地理空间大数据转向”。这一转向催生了包括全球地理空间地图在内的许多实用方法与工具, 但它们可能会影响基于权利的包容性保护方法, 也就是考虑多元价值与视角的保护实践。我们根据不断积累的文献, 呼吁加强关注全球生物多样性保护绘图所依赖的数据集、方法选择及基本假设。随着保护工作者越来越优先使用大地理空间数据, 他们可能会忘记这些地图仅呈现了部分信息, 限制了认识和表征世界的方式的多样性。通过遥感技术收集的地理空间大数据具有特定的时间和空间背景, 且需要提供相应的政治经济与社会文化背景。此外, 即便在推动数据开放的背景下, 全球绘图工作仍主要由全球北方的研究者主导。我们呼吁保护工作者避免盲目追求更多的数据, 而是应审慎地考虑地理空间大数据的具体背景, 从而推动领域实现社会公正及生态保护效益。**【翻译:胡怡思;审校:聂永刚】**

## INTRODUCTION

Conservation science has increasingly adopted digital technological advances in recent decades, employing big data to identify planetary environmental changes (Runting et al., 2020), monitor wildlife populations (Tuia et al., 2022), and improve understanding of human interest in nature (e.g., conservation culturomics [Ladle et al., 2016]). Despite wide usage of the term, there is no precise definition for *big data*. It is generally characterized by large volumes of data that are generated or made available at high frequencies, are of various types and from various sources, are exhaustive (rather than sampled), and often require new tools and massive computational power through cloud and high‐performance computing for analyses. *Big data* also refers to how data are understood and used—that is, they reflect a belief that datafication can capture reality accurately and that human life and the world can be quantified for analyses (Kitchin, 2014; Thatcher, 2014). Although the field of biodiversity conservation has expanded from its biological foundations to include the social sciences and humanities (Bennett et al., 2017), the emerging epoch of faith in data threatens to recenter potentially exclusionary modes of conservation.

Even with the wide range of themes that conservation science now addresses, area‐based approaches remain central to the mission, and there are repeated calls for more spatial data and maps to guide action (Hawkins, 2024; Naidoo et al., 2008; Runting et al., 2020; Schmidt‐Traub, 2021), reflected in the recent proliferation of geospatial analyses and global mapping efforts (Cobb et al., 2024). Technological innovations and use of big data, especially geospatial data, can help reveal planetary‐scale, human‐driven changes in the environment and identify areas of conservation priority, given limited resources. Big geospatial data approaches thus aid conservation decision‐making and enforcement, enhance transparency and monitoring compliance (Runting et al., 2020), and increase the spatial and temporal extent of understanding ecological dynamics and managing protected areas (Marvin et al., 2016).

Despite enthusiasm over big geospatial data's potential, recent studies highlight the potential downsides of their uncritical application (Bennett et al., 2024; Kloppenburg et al., 2022; Pritchard et al., 2022; York et al., 2023). We built on the emerging field of data justice, which foregrounds the discourses, practices, and infrastructures around big data and their potential harms and injustices (Dencik et al., 2022; Nost & Goldstein, 2022), and drew on critical data and critical GIS studies to examine whether and how big geospatial data may enable or hinder effective and just conservation science, policy, and practice. We considered whether diverse ways of seeing, thinking, and understanding (IPBES, 2022) might be lost when the nature to be protected becomes primarily perceived through pixels and bytes of data visualized on a screen (Dalton & Thatcher, 2014). In the same vein, problem framings could become limited to that which can be measured, quantified, and classified through digital technologies, which could reinforce power imbalances and dominant discourses (Rajão, 2013). Although novel injustices may arise from the use of big geospatial data, the problem in particular is how big data thinking risks perpetuating long‐standing, systemic injustices that conservation has been working to address (Archer et al., 2022; Carmenta et al., 2023; Raymond et al., 2022).

## BIG GEOSPATIAL DATA IN CONSERVATION

### Shift toward use of big geospatial data

Historically, conservation biology was largely concerned with population biology, natural resource management, and macro ecological processes of biodiversity distribution (Soule, 1985). Early conservation efforts relied primarily on localized field research and expert opinion built on years of lived experience in specific geographies or work on specific taxa. The end of the Cold War precipitated the nonmilitary use of satellites and imagery, and remote sensing became increasingly linked to Earth observation for environmental monitoring by state and nonstate actors (Olbrich, 2019). Satellite images were (and sometimes still are) perceived as objective (Bennett et al., 2024; Litfin, 1997), covered large spatial extents beyond what is possible through field or manual methods, and provided somewhat regular temporal updates. Satellite navigation systems, such as the U.S. Global Positioning System (GPS), and increased technology access for wider populations also boosted the collection of global biodiversity data, enabling cross‐taxonomic analyses at regional and global scales (Heberling et al., 2021), although global data retain geographic and taxonomic biases (Donaldson et al., 2017; Troudet et al., 2017). These advances in digital technology and computing power have enabled the development of global, high spatial and temporal resolution geospatial data relevant to biodiversity.

Although field training remains central in conservation‐related degrees (Slater et al., 2024), at graduate levels, GIS and data analytical skills are increasingly perceived as important and necessary, perhaps more so than taxonomic identification and ecological sampling skills (Bernd et al., 2017; Tuia et al., 2022). This institutional de‐emphasis of ecological fieldwork is reflected in funding reductions to field stations, despite their continued centrality to ecological fieldwork and conservation science, including in validating satellite‐derived data (Eppley et al., 2024). Similarly, major conservation‐focused nongovernmental organizations (NGOs) have embraced the production and use of global maps (Brooks et al., 2006; Redford et al., 2003) to identify priority areas and are investing resources into their geospatial analysis capacity—for example, The Nature Conservancy has at least 1800 staff working with geospatial data (Ferdana et al., 2024).

This shift toward big geospatial data may be facilitated by its perceived policy relevance, publishability (Wyborn & Evans, 2021), and the relative ease and low cost of undertaking such analyses within Global North institutions, thanks to the availability of free and open‐source tools (e.g., R, Python, and QGIS) and open‐access datasets (Chavan & Penev, 2011; Turner et al., 2015). These types of analyses contrast with the time‐consuming nature of designing field experiments and collecting in situ data, which necessitates equipment, travel, and other logistical costs and can be upended by unforeseen circumstances, such as the 2019 global COVID pandemic. It may also be seen as a way to avoid the unethical practices associated with parachute science, where (usually) Global North researchers conduct research in Global South contexts without sufficiently engaging local researchers or communities (Baker et al., 2019; De Vos & Schwartz, 2022; Trisos et al., 2021). We posit that these broader trends have facilitated the big geospatial data turn in conservation.

### Mobilizing big geospatial data for better conservation

Protected areas are the primary means of enacting conservation on the ground, but since the 2000s, there have been debates over their effectiveness and concerns that their designations were more politically influenced than biologically based (Chapin, 2004; Harris & Hazen, 2005). The imperative to assess and demonstrate effectiveness of protected areas in mitigating biodiversity loss and representing biodiversity and the perceived need to use limited funds wisely (Bottrill et al., 2008) arose as biodiversity loss began to be recognized as a global problem requiring global solutions. These imperatives and perceptions have contributed to a burgeoning focus on global conservation prioritization and impact evaluation.

Prioritizations have focused on the irreplaceability of biodiversity, such as species, phylogenetic, functional, and ecological diversity (e.g., Global 200 priority ecoregions [Olson & Dinerstein, 2002]); high levels of vulnerability to threats (e.g., biodiversity hotspots [Myers et al., 2000]); gap analyses to mitigate future threats (e.g., Lee & Jetz, 2008); intactness of habitats (e.g., last of the wild [Allan et al., 2017]); and nature's contributions to people (e.g., critical natural assets [Chaplin‐Kramer et al., 2023]). These prioritizations have added to the growing number of global maps of land cover (e.g., global forest change [Hansen et al., 2013]); human pressures (e.g., anthropogenic threat complexes [Bowler et al., 2020]); ecosystem state (e.g., forest landscape integrity index [Grantham et al., 2020]); economic cost of agricultural lands (e.g., Naidoo & Iwamura, 2007); and other biophysical factors (e.g., WorldClim [Hijmans et al., 2005]).

To increase accessibility and in line with FAIR (findable, accessibility, interoperability, and reusability) principles (Wilkinson et al., 2016), many of these datasets are published open access. Some are also available on Google Earth Engine to provide computational capacity to more users (Gorelick et al., 2017), and data platforms on which users can view and perform simple queries on the data have emerged to allow users to monitor threats in nearly real time (e.g., Global Forest Watch [https://www.globalforestwatch.org/], Global Fishing Watch [https://globalfishingwatch.org/]). Other tools, such as Southeast Asia Climate and Nature‐based Solutions Coalition Tool (https://nbstool.scenecoalition.org/), allow local NGOs and communities to develop their own projects. Particularly in conservation and development, international NGOs have a strong influence on academia and are also influenced by academic discourses and are able to scale up practices on the ground (Fisher, 1997; Kiik, 2019). The development of big geospatial data and tools for their analyses, especially by international organizations, can greatly influence uptake and reach of big geospatial data.

### Representing people in big geospatial data for better conservation

Efforts (predominantly in geography) to link remote sensing with social sciences have flourished over the past decades (Liverman & Cuesta, 2008; National Research Council, 1998) and have influenced geospatial applications in conservation research and practice. Recent Intergovernmental Science–Policy Platform on Biodiversity and Ecosystem Services reports emphasize the diversity of values that underpin conceptualizations of human–nature relationships in various sociocultural contexts, highlighting the need for coproduction and engagement with a fuller spectrum of values for more effective conservation (IPBES, 2022). The proliferation of data and the ability to combine non‐geospatial social, economic, and political data with geospatial information have thus enabled global maps to recognize and make visible new actors.

For example, the production of the Indigenous Peoples’ lands map (Garnett et al., 2018) has facilitated inclusion of these lands in global research on primate conservation (Estrada et al., 2022), comparisons of the lands’ relative effectiveness in reducing deforestation (Sze et al., 2022), and identifications of threats to these lands by industrial development (Kennedy et al., 2023). Instead of solely focusing on biodiversity, Chaplin‐Kramer et al. (2023) used at least 37 global spatial datasets to identify critical natural assets vital to human well‐being, such as data on anthromes (Ellis & Ramankutty, 2008), linguistic diversity (Eberhard et al., 2020), and rural and urban populations (Cattaneo et al., 2021). Increasing Internet and smartphone penetration in rural communities is also facilitating Indigenous Peoples’ and local communities’ efforts to counter‐map their territories and socioecological systems and thus monitor biodiversity, identify illegal activities, and gain land rights recognition (Ryan et al., 2023; Sauls et al., 2023). These maps have become important tools for recognizing and communicating about communities’ efforts to assert land and territorial rights.

Given rapid technology developments, it is likely that future global maps will increasingly incorporate data from sources other than satellites, such as acoustic monitoring, environmental DNA, drones, locational data from photos, and citizen scientists. These data can reveal biodiversity status or threats with greater accuracy and at higher temporal resolutions, and some such maps are already available at regional scales (e.g., acoustic indices‐based ecological integrity of Andes in Colombia [Sánchez‐Giraldo et al., 2021] and threats to ecological integrity in Iceland based on tourist photos [Hale, 2018]).

## PROBLEMATIZING CONSERVATION'S GEOSPATIAL TURN

### Methodological challenges for global conservation

**FIGURE 1:**
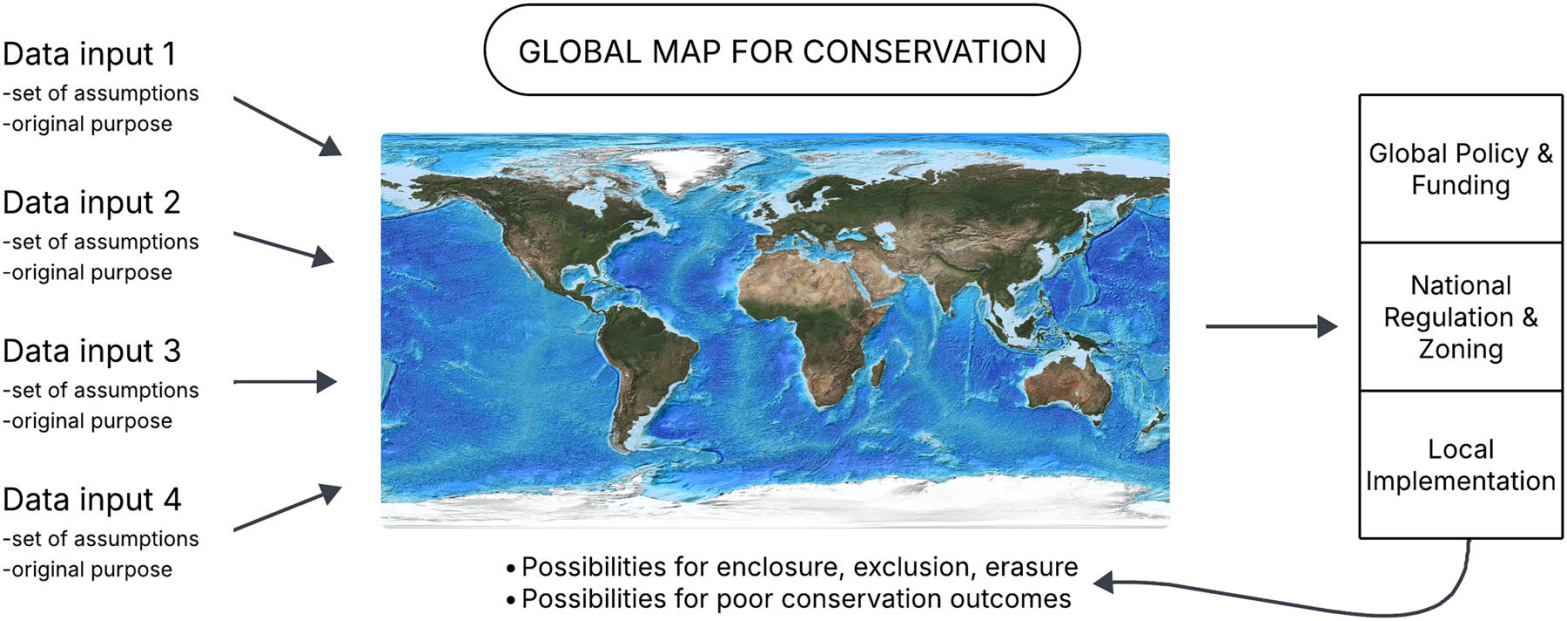
How the use of big geospatial data in global mapping for conservation purposes has social and ecological implications at various scales. Data inputs 1–4 (e.g., land cover, human population density, and species distribution models) are integrated, which could mean a layering of different assumptions and different estimated outcomes based on the underlying spatial and temporal data that could be at different resolutions. Possible outcomes are shown under the map. Base map by Kevin M. Gill, CC BY 2.0.

The construction of early global biodiversity datasets involved compiling records and databases across countries and regions, requiring interpretation over different naming conventions and classification systems, and standardizing or homogenizing for integration (Bowker, 2000). Metadata and data quality standards emerged to make data compatible and shareable across different data collectors and users, even though this could obscure differences on the ground (Edwards et al., 2011). The difficulties presented by combining data persist and are perhaps exacerbated by the proliferation of datasets in conjunction with interdisciplinary efforts in conservation to incorporate socioeconomic and ecological data. These difficulties are especially evident in global mapping efforts that depend on data at different scales and temporal resolutions and that are based on collection and generation methods that differ (e.g., crop yield maps based on census statistics, which are still widely used [Monfreda et al., 2008]). A basic tenet of science is to ensure that data are fit for purpose, but in the era of big geospatial data, datasets are often reused for other purposes. This contributes to the difficulties of determining original sources and relevant metadata that can make fitting the data to the purpose harder (Figure 1). Resultant maps have a veneer of reality in that they provide results for every place on Earth and address the problem at hand at a global scale. However, their use to inform action or policy debates and priority‐setting exercises at other scales could be problematic given the uncertainties of the underlying data, which can lead to poor ecological and social outcomes.

Even without incorporating social data, ecological and biological data are subject to human interpretation and assumptions in decision‐making, some of which may be unconscious. Land‐cover maps derived from satellite imagery are widely used in conservation and perceived to be universally applicable; yet, these land‐cover classifications are often questioned by local communities due to differences in how they perceive the landscape (Herrmann et al., 2014; Robbins, 2003). Most global forest maps are satellite derived, relying on remote sensing researchers working with spectral reflectance indices and supervised, automated classification (e.g., Hansen et al., 2013) to produce information on tree canopy cover in each pixel. Conservation researchers then decide on a threshold value for categorizing a pixel as forested. Yet, how forests are categorized reflects underlying values and can marginalize local knowledge (Cheyns et al., 2020).

Interpreting land use from land cover is even more subjective. For example, distinguishing pastoral land use from grassland land cover that is assumed to be degraded and in need of restoration is tricky and can result in inappropriate recommendations for restoration that can negatively affect, often marginalized, local communities if implemented (Fleischman et al., 2022; Schultz et al., 2022). Remote sensing may not easily detect some land uses, as in the case of changes in vegetation structure in Indigenous‐modified forests in Panama (Runk et al., 2010). Understanding the sources of data and differences in interpretation of definitions and categories thus affects how data are represented, how cause and effect are interpreted, and what management interventions are required and where (Liverman & Cuesta, 2008). Instead of interpreting land use from remotely sensed data from the top‐down, first identifying key land‐use activities and actors to characterize land systems may provide more appropriate and suitable land‐use mapping (Pratzer et al., 2024).

Methodological choices are well known to be critical to determining the final composition—and thus potential meanings—of a map (Lecours, 2017; Monmonier, 2018). The datasets researchers choose and the underlying quality and scale of those data can produce dramatically different maps of the same phenomena. Identifying land use and land cover is crucial to determining habitat availability for species. Although these maps themselves are based on various assumptions, in mapping possible species distribution based on habitat suitability, it is sometimes decided to, for example, omit all agricultural land, regardless of the type or intensity of agriculture (e.g., Strassburg et al., 2020). Maps may be produced by allocating fractional estimates to each pixel (e.g., harmonized above‐ and below‐ground biomass carbon density [Spawn et al., 2020]) or by assigning the mean value to all pixels (e.g., potential carbon sequestration [Strassburg et al., 2020]). These methodological differences may be due to data constraints, but they produce different outcomes that have different implications for different end users. Other methodological assumptions may result from unconscious biases, such as assuming that human influences on the environment must necessarily be negative or universalizing categories of land uses and land covers. Although these examples reflect common problems in data analyses, as the volume of data increases and gets more exhaustive, they may erroneously promote the illusion of more accurate portrayals of reality.

### Underlying challenges of the big geospatial data approach

Many prevailing tensions concerning the use of global geospatial data revolve around the assumption that these data are objective (Hazen & Harris, 2007) or, more subtly, that through the production of a global map, problems are framed objectively (Malavasi, 2020). The ability of digital technologies to capture increasing quantities of high‐quality data furthers the impression of objectivity and supports claims that they provide a robust evidence base for conservation interventions. Further, although these maps are at the global scale, they are mostly produced by Global North researchers and institutions, given their greater funding and access to data, software, and relevant training than researchers based in the Global South (Cobb et al., 2024; Redford et al., 2003). This results in maps that propagate particular ways of understanding the world (ontology) and knowledge and knowledge production (epistemology) (Moon & Blackman, 2014) (i.e., ontologies and epistemologies of a narrow set of values) (Raymond et al., 2023). The visibility and prominence of maps can sideline written documents and oral traditions grounded in specific places, potentially obscuring histories of occupation, ethnic cleansing, and other social injustices.

Using big geospatial data often means overlaying and aggregating (or subtracting) different maps to identify conservation priorities (e.g., Rayden et al., 2023; Simmonds et al., 2023). These prioritization exercises may draw on inputs that primarily reflect economic market costs and values. These inputs are incongruous with the incommensurable diversity of values, the ways in which they are articulated, and the worldviews in which they exist (Raymond et al., 2023), particularly with Indigenous Peoples and local communities who use and value land in multiple and overlapping ways. Big geospatial data approaches also often omit Indigenous and traditional knowledges and uses of land because they translate poorly to maps. Sometimes these knowledges are intentionally overlooked to protect their epistemological traditions and sacred sites (Bryan, 2009; Rubis & Theriault, 2020; but see Pearce & Louis [2008] on Indigenous cartography). Priorities for global conservation thus depend on specific ontological frames, rather than universal conditions.

Academic research is increasingly acknowledging the value and importance of Indigenous‐led counter‐mapping, participatory conservation, and coproduction to ensure more just and often more effective outcomes (Newing et al., 2024; Sauls et al., 2023). These approaches can also be of great utility in shifting the power balance toward marginalized communities. However, they do not necessarily scale well to the global level. Particularly because such approaches are attempts to improve equity through the accepted means of the dominant knowledge production and sociopolitical system, they are constrained to what can be achieved given the limits of the system (Bryan, 2009; Sletto et al., 2021). By uncritically promoting greater mapping efforts to more effectively achieve conservation outcomes, the inclusion of usually marginalized knowledge can instead become extractive rather than coproductive. Rather than countering colonial legacies of cartography, it may add to them. Maps were crucial to European processes of colonization, from navigation to resource exploitation (Eichhorn et al., 2020). Ecology and natural history, the foundational disciplines of conservation, have clear roots in colonial exploitation, and the imperatives of global prioritization exercises highlight the ongoing power asymmetries between those who identify these priorities and those who live in spaces that become prioritized.

### Centering the ethics of big geospatial data use

This history of colonization and conservation is of great concern because many of the digital technological advances lauded as conservation aids relate to trends toward greater surveillance and control (Adams, 2019; Nadim, 2016; York et al., 2023). Global Fishing Watch provides unprecedented tracking of fishing fleets’ movements and demonstrates where illegal fishing has occurred and by which flag states, but it entails enhanced surveillance that reinforces existing unequal Global North–Global South political and economic relations and power structures (Drakopulos et al., 2023). There is, appropriately, a growing concern about the ethics of and injustices associated with the monitoring and surveillance technologies in conservation (Millner et al., 2024; Sandbrook et al., 2018; Young et al., 2022). Remotely sensed data collection methods remove socioecological context from data and involve a distancing between researchers and the places (and inevitably people) they study. This distance may facilitate coercive conservation governance. The production of seemingly authoritative maps is used to inform decisions, rather than decisions being made through processes that consider a full, coproduced understanding of socioecological systems (Kiggell, 2021; Millner et al., 2024).

Ironically, the distance between researchers and their study areas created by the use of remotely sensed data can be compounded by open science. Open‐access maps facilitate their use by other researchers, but sufficient attention may not be paid to the production process. This means geospatial products can be inappropriately repurposed for monitoring and enforcement. Goldstein (2020) shows how the global MODIS fire hotspot maps applied to Indonesia's peatlands are fraught with inaccuracies and distortions due to the satellite infrastructure and the nature of peat fires. Yet, they are used, along with problematic land tenure maps, to identify probable arsonists to assign blame. The use of global maps at local levels, though possible, may not always be appropriate because the scale at which data are collected and processed may not fit the scale at which it is used. For example, although useful for showing global changes in tree cover, the Global Forest Change data overestimate forest cover in Gabon and thus, for Gabon, require calibration with sample reference data (Sannier et al., 2016).

As such, providing user‐friendly platforms from which to access and analyze global geospatial data may not necessarily achieve effective or just outcomes. Users may not fully understand—or receive sufficient information to understand—the processes behind the data presented (Lecours, 2017). More importantly, they may be constrained by having to work with the limited information provided and within the dominant framing and objectives of the organization behind the platform. Efforts to standardize data, data types, and databases for interoperability and to facilitate greater uptake also have drawbacks in that they homogenize and limit the diversity of views and information (Bowker, 2000). Parachute science notwithstanding, traveling to field sites for data collection allows researchers to gain insights into the local socioecological system and the human communities living in or around their field sites. Site visits provide the opportunity to understand the ecological processes and outcomes measured as mediated by other social, political, or economic factors. Collaborative research grounded in place‐based engagement is necessary—with remote sensing as well as on its own—to ensure more just conservation practice (Robinson et al., 2023).

Further tensions exist between making data open and adhering to FAIR principles and respecting data sovereignty and consent. Guidelines have been developed to respect Indigenous Peoples’ rights and sovereignty concerning themselves and their environment (CARE [collective benefit, authority to control, responsibility, ethics] principles [Carroll et al., 2021]). de Lima et al. (2022) highlight structural and economic inequities regarding open data requirements and those who collect field data, data that are often required for validating satellite data. Nonetheless, most global geospatial data are currently derived from satellites, which pose particular difficulties given their broad coverage and the lack of free, prior, and informed consent by any of the world's surveilled populations. Although earlier satellites could not capture information at the level affecting individuals (Landsat 1‐3, launched in 1972, had a spatial resolution of 80 m), more recent commercial very‐high‐resolution (∼30 cm) and ultrahigh‐resolution (∼10 cm) satellites could pose greater personal harm, particularly when combined with image‐processing analyses and other data (Rapp & Santos, 2019). Despite emerging calls for more ethical remote sensing (Bennett et al., 2024), these concerns are likely not being considered adequately by conservationists (York et al., 2023).

Although less thoroughly assessed in the context of conservation, the rise of corporate due diligence and environmental, social, and governance compliance auditing, especially by private consultancies, may also lead to risks in the use of big geospatial data. Here, the potential harm is 2‐fold. First, data and information that are often freely available become privatized and sold for profit, without benefit to the data providers or communities the data are about (Florio, 2024). Second, inadequate understanding of local contexts may lead to the data being misinterpreted and misused in ways that cause injustice to places and people (even if well intentioned) and that lack accountability.

## BUILDING BETTER GEOSPATIAL PRACTICE IN CONSERVATION

All the conservationists we know (and we count ourselves among them) have good intentions. These intentions are reflected in their desire to reduce negative human impacts on other species and efforts to make conservation more inclusive and fair, including through the Global Biodiversity Framework (Archer et al., 2022; Raymond et al., 2022). Yet, the training most conservationists receive—largely in the biological sciences (Slater et al., 2024)—and the problem framings and narratives they inherit can contribute to problematic conservation interventions. In particular, implicit framing of human–nature divide pervades mainstream conservation training. For example, persistent narratives of population growth and livestock expansion as threats to biodiversity in the Bale Mountains of Ethiopia legitimize efforts to resettle the people living there, despite evidence that population growth and livestock do not threaten biodiversity (Chignell & Satterfield, 2023). The idea that more data are better and necessary for effective conservation may direct more funding and research to data‐driven and digital‐technology‐enabled approaches (e.g., Bezos Earth Fund's AI Grand Challenge for Climate and Nature [https://www.bezosearthfund.org/news‐and‐insights/phase‐i‐grants‐ai‐grand‐challenge‐climate‐nature]) and detract from processes that could achieve more just social and ecological outcomes.

Users of big geospatial data should consider wider political, economic, and sociocultural contexts, especially in problem formulation. For example, in counterfactual approaches to evaluating effectiveness of protected areas, many studies evaluate only areas listed in the World Database of Protected Areas as protected. Yet, with the increasing diversity of conservation solutions, including wildlife corridors, landscape conservation planning, private or community‐based reserves, and Indigenous territories (Qin et al., 2024), this may produce erroneous conclusions with repercussions for nontraditional conservation actors. Beyond simply applying more sophisticated analytical techniques with more and assumed better data, conservation demands more rigorous approaches, incorporation of plural methodologies, and consideration of structural forces, including underpinning colonial dynamics (Coetzee & Gaston, 2021; Frame, 2022; Holden et al., 2024).

Instead of uncritically embracing the use of global maps or not using them at all, we encourage conservationists to be aware of and clear about what the data they create or use can and cannot do and consider how methodological tweaks might change their results and the potential consequences of their research on communities where conservation is implemented. Working across disciplines and with local communities coproductively can help generate these answers. We reiterate that, at a minimum, researchers should ensure good data management and metadata curation, making information about the decisions and assumptions made in data processing and cleaning explicit, preferably in the main text of publications (e.g., Bennett et al., 2024; Pritchard et al., 2022; Robinson et al., 2023; York et al., 2023). These would facilitate other researchers in understanding the maps being produced and how best they can use them.

## CONCLUSION

The big geospatial data turn in conservation is arguably redefining how conservation is enacted. Global maps are important for understanding the pressures on and state of biodiversity at the planetary scale. They reveal the connections between where demands for resources are and where these impacts are felt. Yet, conservationists need to bear in mind that any given map reveals only a partial view and is inevitably a simplification of reality.

We suggest that big geospatial data follow the law of diminishing returns. The proliferation and accessibility of increasing quantity and quality of data—particularly, the aggregation of such data—allow one to examine the world in ways that were previously not possible (Runting et al., 2020). Yet, past a certain point, the sheer quantity of data being collected no longer provides additional or novel insights. Instead, it narrows potential solutions to those measurable and solvable within the dominant framing. Apart from limiting the ways in which conservationists understand problems and propose solutions (Kloppenburg et al., 2022), big data computation methods also exert a high cost on the environment (Jay et al., 2024). Critically considering the utility of additional data collection at ever finer scales, being frank about their use and usefulness, and recognizing that understanding of any problem is never complete or representative of all stakeholders will maximize the benefits of their use and minimize potential harms.

In many ways, the role of global maps in conservation brings up 2 old questions: how do humans and nature relate to each other and what is the goal of conservation (Redford & Sanderson, 2000)? We argue that the big geospatial data turn reinforces separation between humans and nature, possibly taking both humans and nonhuman nature out of conservation. What researchers choose to study and what scientific articles are about are “reflections and vehicles for promoting our frames and agendas,” for disseminating what we think conservation should be and do (Massarella et al., 2021). Given the convergence of global targets, technological innovation, and radical reform that conservation is contemplating (Corson & Campbell, 2023), we believe that rather than a blind hype of collecting more data, more and careful attention should be paid to considering how to develop geospatial research, data, and indicators that support Indigenous Peoples’ and local communities’ rights and advance alternative, anticolonial, and socioecologically just visions of conservation—including by consciously and consistently questioning one's own practices.

## AUTHOR CONTRIBUTIONS

Jocelyne Shimin Sze and Laura Aileen Sauls conceived the concept for the manuscript. Jocelyne Shimin Sze wrote the first draft, and both authors contributed to editing and finalizing the manuscript.
